# Response to Letter to the Editor: “Water Supplementation Reduces Copeptin and Plasma Glucose in Adults with High Copeptin: The H_2_O Metabolism Pilot Study”

**DOI:** 10.1210/clinem/dgz085

**Published:** 2019-10-30

**Authors:** Sofia Enhörning, Olle Melander

**Affiliations:** 1 Department of Clinical Science, Lund University, Skåne University Hospital, Malmö, Sweden; 2 Department of Endocrinology, Skåne University Hospital, Malmö, Sweden; 3 Department of Internal Medicine, Skåne University Hospital, Malmö, Sweden

We thank Dr Struja et al. for the comment on our paper. They call the finding of copeptin reduction following addition of 1.5 L of water in low water drinkers “a self-fulfilling prophecy”. We are aware of the physiological feedback mechanisms of the vasopressin system, which was the basis for doing the study. In contrast to what Struja et al. argue, there are not many publications testing if and how much copeptin or vasopressin is reduced after adding moderate amounts of water in healthy adult subjects, and our paper was the first to do so in subjects with hard evidence of low water intake (low urine volume, high urine osmolality and high copeptin). We agree with the conclusion that the clear and substantial reduction of copeptin proved that most of the patients did adhere to the protocol and increased their daily water intake as prescribed. This is important information in a pilot study preceding a large and costly intervention study. As clinicians, we are well aware that good adherence is not to be taken for granted.

Moreover, criticism is being raised against the hypothesis that water may reduce diabetes risk, without referring to any data supporting that criticism. The Diabetes Prevention Program outcomes study, which is cited, did not study the effects of water. We agree that the planned large randomized water intervention study will shed more light on the question (Clinical Trial registration number: NCT03422848).

Whether or not that trial will show effects of increased water intake on fasting glucose level (primary endpoint) in low drinkers, the study is well powered as it can detect down to a 0.14 mmol/L difference between water and control therapy (800 subjects will be randomized). We do not believe that a smaller effect size on fasting glucose than 0.14 mmol/L is clinically relevant, and thus we do not agree that the planned study is underpowered.

Finally, the concern of hyponatremia risk as a result of increasing water intake by 1.5 L per day in low water drinkers lacks scientific evidence. Apart from “common sense” saying that adding 1.5 L of water in low water drinkers appears to be one of the safest interventions one can undertake, the pilot study did not observe one single case of hyponatremia. Furthermore, two previous randomized studies investigated the effect of 1.5 L of increased water intake per day during 6 and 12 months respectively in individuals at risk for hyponatremia, ie, in an elderly population (1) and a population with chronic kidney disease (2), without observing any reduction in sodium concentration due to increased hydration.

## Additional Information

***Disclosure Summary:*** S.E. has accepted conference fees from Danone Research. O.M. has received a research grant and consultancy fee from Danone Research. The authors report no other conflicts of interest in this work.
